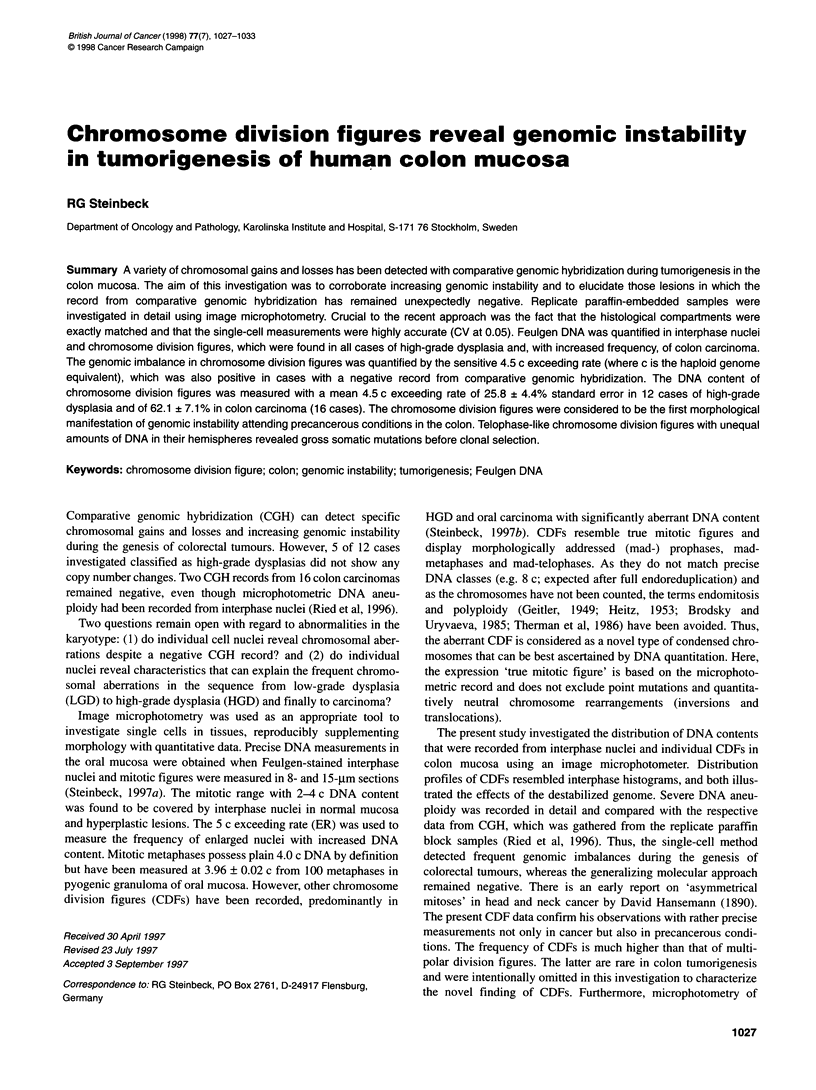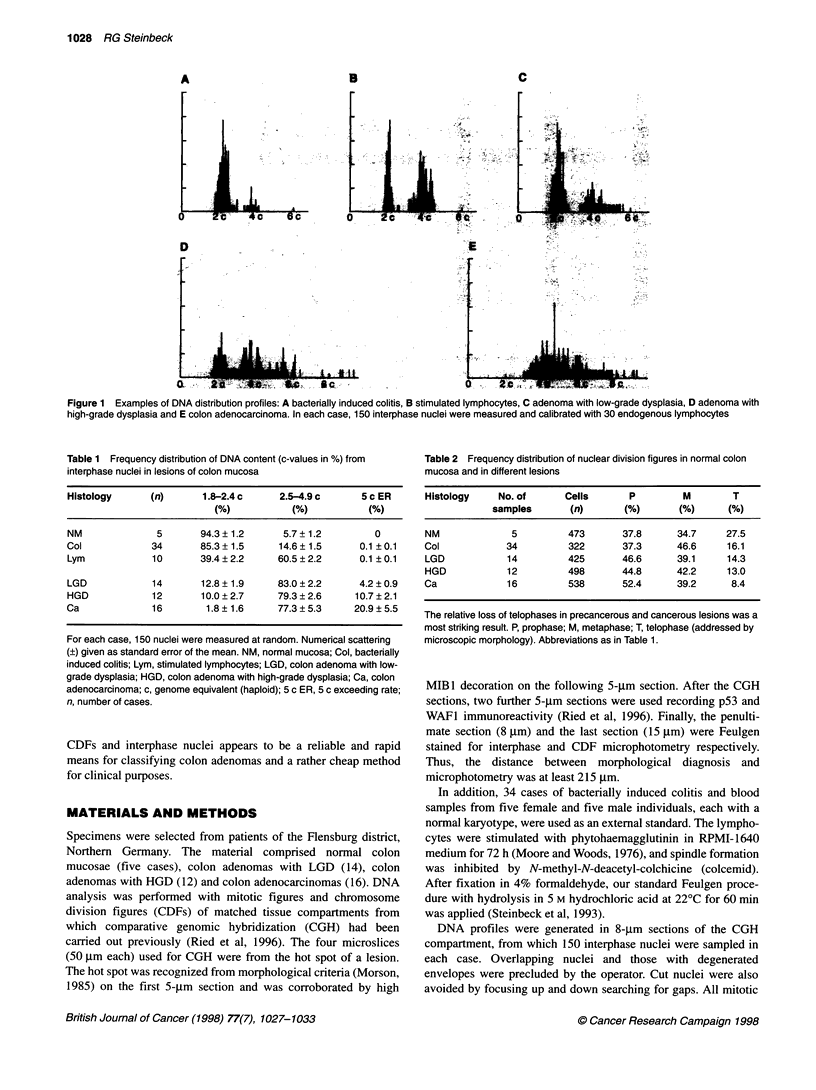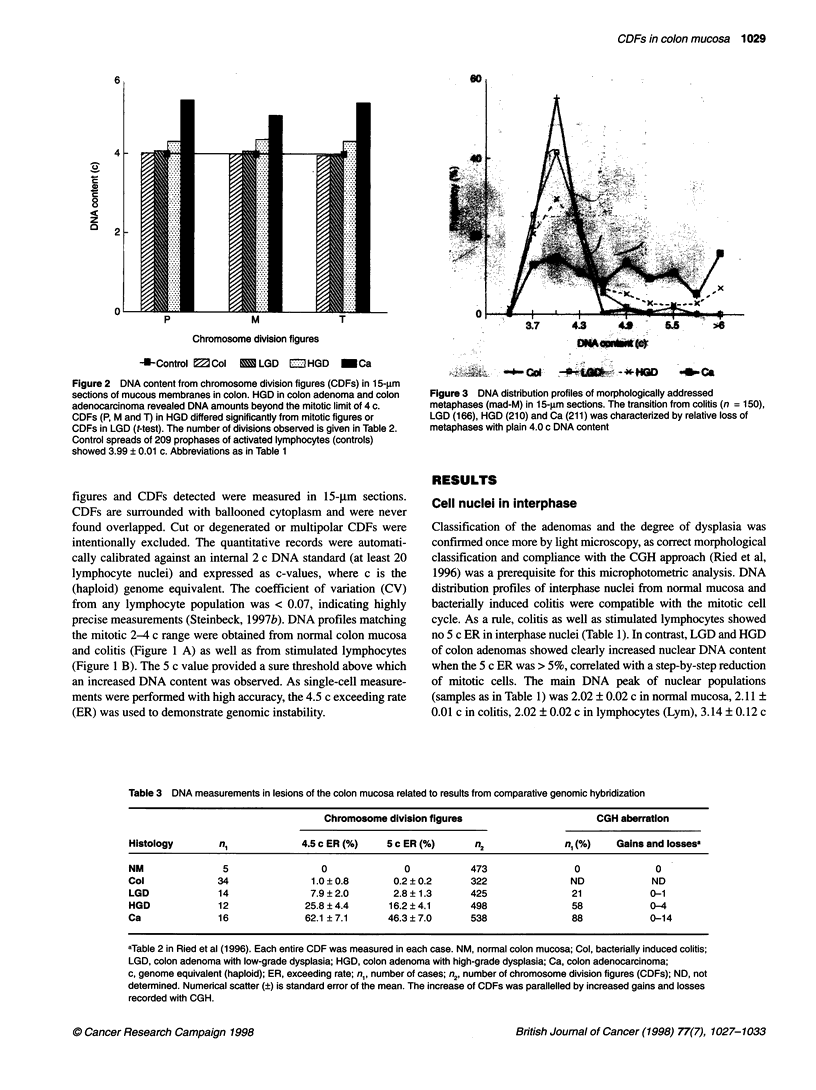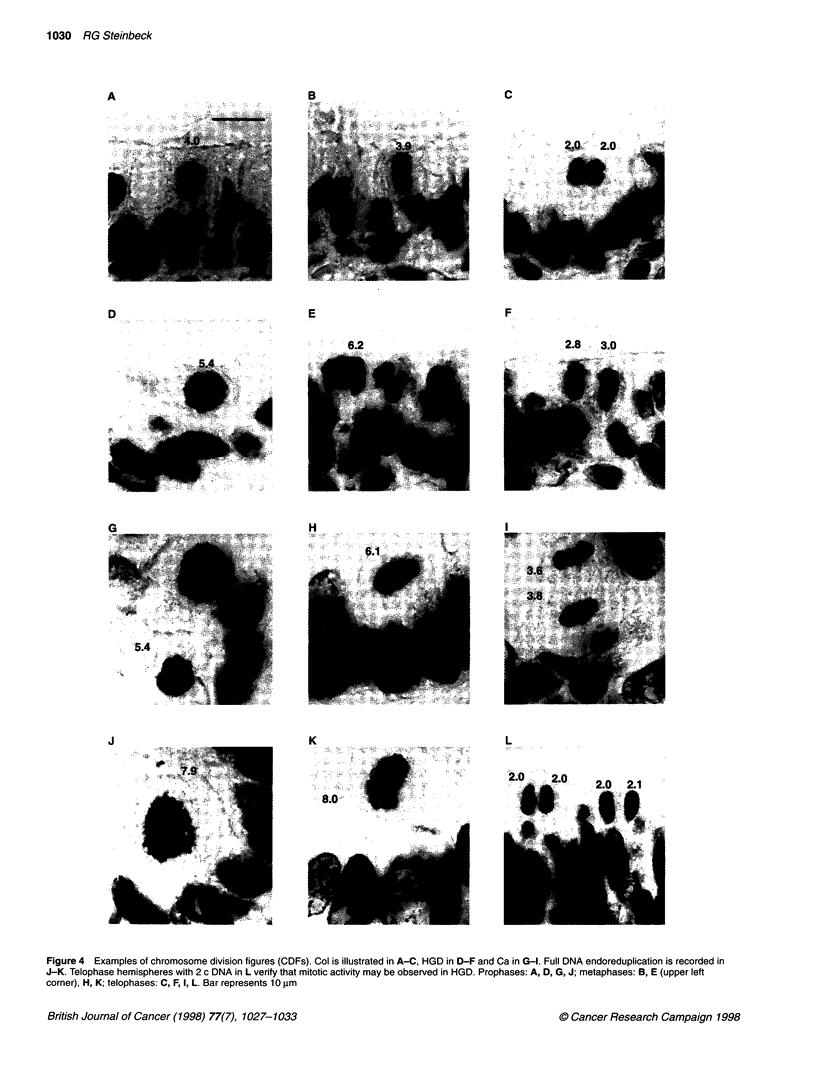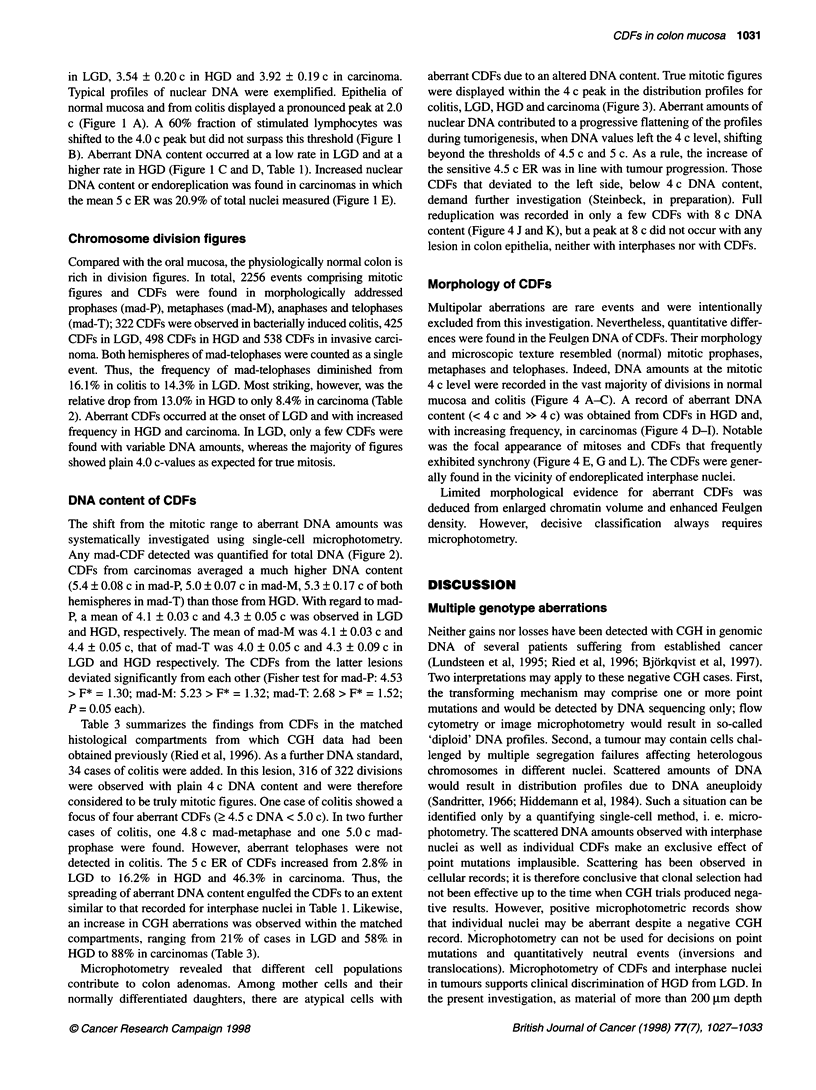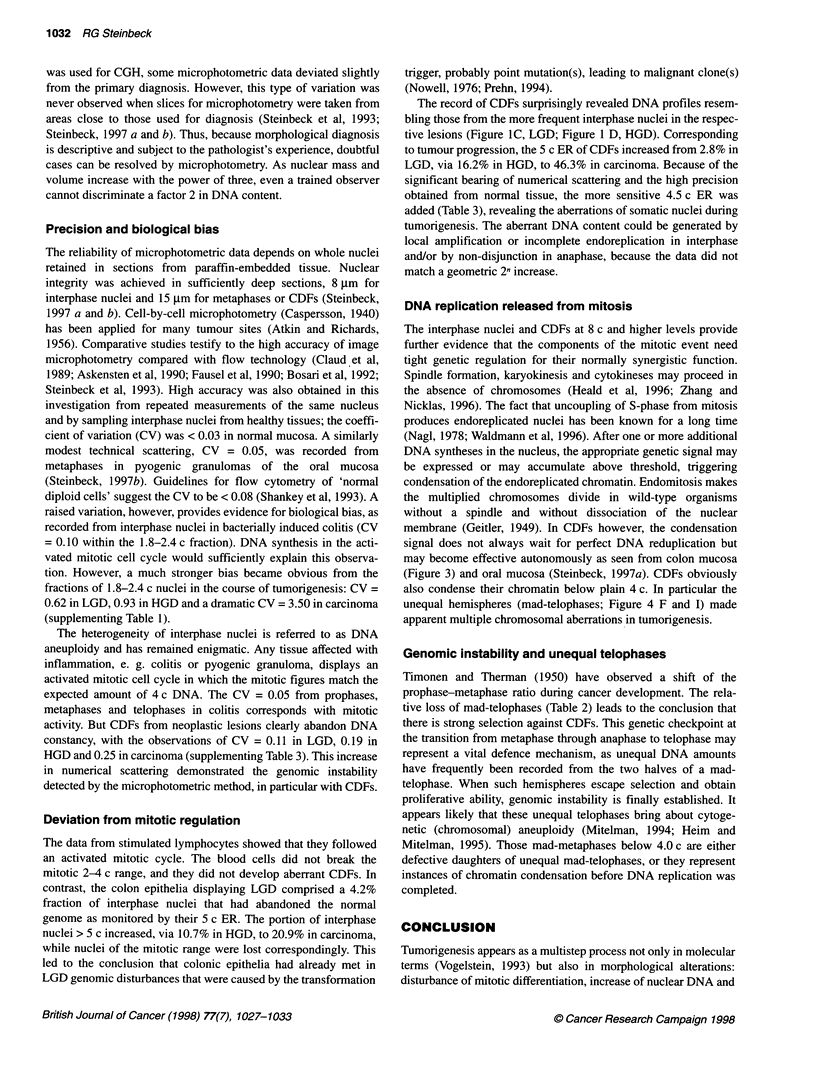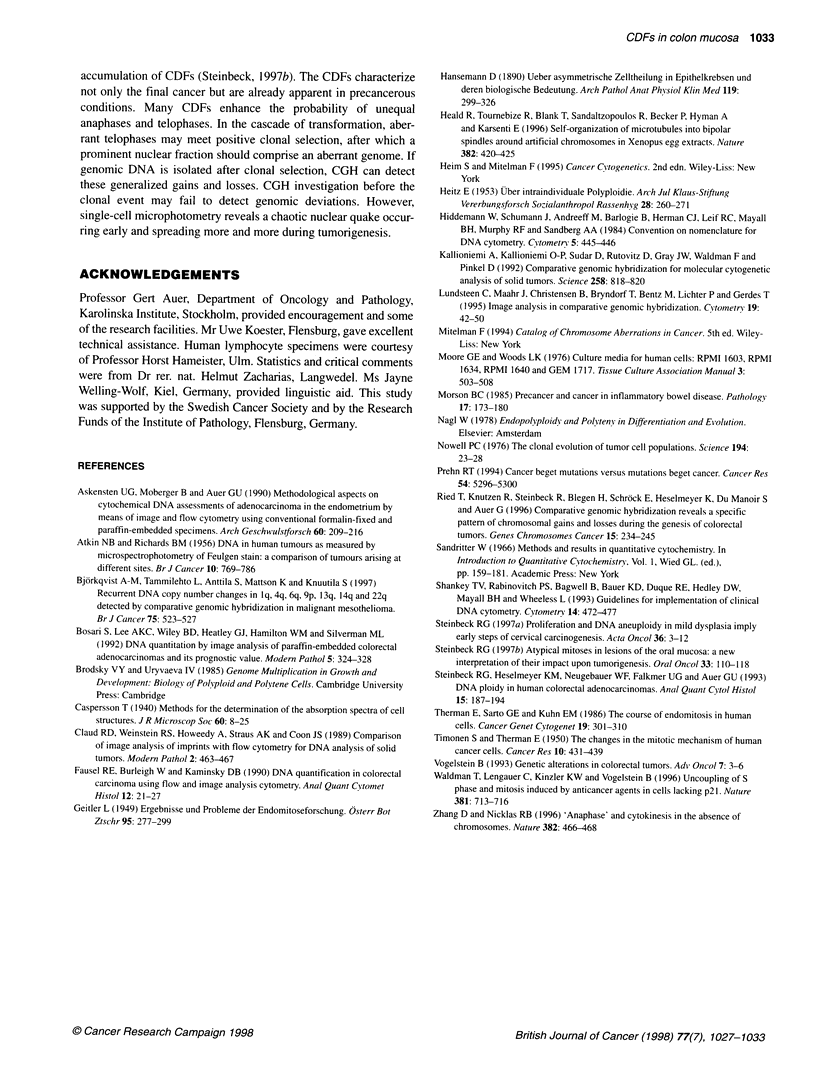# Chromosome division figures reveal genomic instability in tumorigenesis of human colon mucosa.

**DOI:** 10.1038/bjc.1998.171

**Published:** 1998-04

**Authors:** R. G. Steinbeck

**Affiliations:** Department of Oncology and Pathology, Karolinska Institute and Hospital, Stockholm, Sweden.

## Abstract

**Images:**


British Journal of Cancer (1998) 77(7), 1027–1033
© 1998 Cancer Research Campaign

### Chromosome division figures reveal genomic instability in tumorigenesis of human colon mucosa

**RG Steinbeck**

Department of Oncology and Pathology, Karolinska Institute and Hospital, S-171 76 Stockholm, Sweden

**Summary** A variety of chromosomal gains and losses has been detected with comparative genomic hybridization during tumorigenesis in the colon mucosa. The aim of this investigation was to corroborate increasing genomic instability and to elucidate those lesions in which the record from comparative genomic hybridization has remained unexpectedly negative. Replicate paraffin-embedded samples were investigated in detail using image microphotometry. Crucial to the recent approach was the fact that the histological compartments were exactly matched and that the single-cell measurements were highly accurate (CV at 0.05). Feulgen DNA was quantified in interphase nuclei and chromosome division figures, which were found in all cases of high-grade dysplasia and, with increased frequency, of colon carcinoma. The genomic imbalance in chromosome division figures was quantified by the sensitive 4.5 c exceeding rate (where c is the haploid genome equivalent), which was also positive in cases with a negative record from comparative genomic hybridization. The DNA content of chromosome division figures was measured with a mean 4.5 c exceeding rate of 25.8 ± 4.4% standard error in 12 cases of high-grade dysplasia and of 62.1 ± 7.1% in colon carcinoma (16 cases). The chromosome division figures were considered to be the first morphological manifestation of genomic instability attending precancerous conditions in the colon. Telophase-like chromosome division figures with unequal amounts of DNA in their hemispheres revealed gross somatic mutations before clonal selection.

**Keywords:** chromosome division figure; colon; genomic instability; tumorigenesis; Feulgen DNA

Comparative genomic hybridization (CGH) can detect specific chromosomal gains and losses and increasing genomic instability during the genesis of colorectal tumours. However, 5 of 12 cases investigated classified as high-grade dysplasias did not show any copy number changes. Two CGH records from 16 colon carcinomas remained negative, even though microphotometric DNA aneuploidy had been recorded from interphase nuclei (Ried et al, 1996).

Two questions remain open with regard to abnormalities in the karyotype: (1) do individual cell nuclei reveal chromosomal aberrations despite a negative CGH record? and (2) do individual nuclei reveal characteristics that can explain the frequent chromosomal aberrations in the sequence from low-grade dysplasia (LGD) to high-grade dysplasia (HGD) and finally to carcinoma?

Image microphotometry was used as an appropriate tool to investigate single cells in tissues, reproducibly supplementing morphology with quantitative data. Precise DNA measurements in the oral mucosa were obtained when Feulgen-stained interphase nuclei and mitotic figures were measured in 8- and 15-μm sections (Steinbeck, 1997*a*). The mitotic range with 2–4 c DNA content was found to be covered by interphase nuclei in normal mucosa and hyperplastic lesions. The 5 c exceeding rate (ER) was used to measure the frequency of enlarged nuclei with increased DNA content. Mitotic metaphases possess plain 4.0 c DNA by definition but have been measured at 3.96 ± 0.02 c from 100 metaphases in pyogenic granuloma of oral mucosa. However, other chromosome division figures (CDFs) have been recorded, predominantly in HGD and oral carcinoma with significantly aberrant DNA content (Steinbeck, 1997*b*). CDFs resemble true mitotic figures and display morphologically addressed (mad-) prophases, mad-metaphases and mad-telophases. As they do not match precise DNA classes (e.g. 8 c; expected after full endoreduplication) and as the chromosomes have not been counted, the terms endomitosis and polyploidy (Geitler, 1949; Heitz, 1953; Brodsky and Uryvaeva, 1985; Therman et al, 1986) have been avoided. Thus, the aberrant CDF is considered as a novel type of condensed chromosomes that can be best ascertained by DNA quantitation. Here, the expression 'true mitotic figure' is based on the microphotometric record and does not exclude point mutations and quantitatively neutral chromosome rearrangements (inversions and translocations).

The present study investigated the distribution of DNA contents that were recorded from interphase nuclei and individual CDFs in colon mucosa using an image microphotometer. Distribution profiles of CDFs resembled interphase histograms, and both illustrated the effects of the destabilized genome. Severe DNA aneuploidy was recorded in detail and compared with the respective data from CGH, which was gathered from the replicate paraffin block samples (Ried et al, 1996). Thus, the single-cell method detected frequent genomic imbalances during the genesis of colorectal tumours, whereas the generalizing molecular approach remained negative. There is an early report on 'asymmetrical mitoses' in head and neck cancer by David Hansemann (1890). The present CDF data confirm his observations with rather precise measurements not only in cancer but also in precancerous conditions. The frequency of CDFs is much higher than that of multipolar division figures. The latter are rare in colon tumorigenesis and were intentionally omitted in this investigation to characterize the novel finding of CDFs. Furthermore, microphotometry of

*Received 30 April 1997*
*Revised 23 July 1997*
*Accepted 3 September 1997*

*Correspondence to:* RG Steinbeck, PO Box 2761, D-24917 Flensburg, Germany

**Figure 1** Examples of DNA distribution profiles: **A** bacterially induced colitis, **B** stimulated lymphocytes, **C** adenoma with low-grade dysplasia, **D** adenoma with high-grade dysplasia and **E** colon adenocarcinoma. In each case, 150 interphase nuclei were measured and calibrated with 30 endogenous lymphocytes

**Table 1** Frequency distribution of DNA content (c-values in %) from interphase nuclei in lesions of colon mucosa

| Histology | (*n*) | 1.8–2.4 c (%) | 2.5–4.9 c (%) | 5 c ER (%) |
|---|---|---|---|---|
| NM | 5 | 94.3 ± 1.2 | 5.7 ± 1.2 | 0 |
| Col | 34 | 85.3 ± 1.5 | 14.6 ± 1.5 | 0.1 ± 0.1 |
| Lym | 10 | 39.4 ± 2.2 | 60.5 ± 2.2 | 0.1 ± 0.1 |
| LGD | 14 | 12.8 ± 1.9 | 83.0 ± 2.2 | 4.2 ± 0.9 |
| HGD | 12 | 10.0 ± 2.7 | 79.3 ± 2.6 | 10.7 ± 2.1 |
| Ca | 16 | 1.8 ± 1.6 | 77.3 ± 5.3 | 20.9 ± 5.5 |

For each case, 150 nuclei were measured at random. Numerical scattering (±) given as standard error of the mean. NM, normal mucosa; Col, bacterially induced colitis; Lym, stimulated lymphocytes; LGD, colon adenoma with low-grade dysplasia; HGD, colon adenoma with high-grade dysplasia; Ca, colon adenocarcinoma; c, genome equivalent (haploid); 5 c ER, 5 c exceeding rate; *n*, number of cases.

**Table 2** Frequency distribution of nuclear division figures in normal colon mucosa and in different lesions

| Histology | No. of samples | Cells (*n*) | P (%) | M (%) | T (%) |
|---|---|---|---|---|---|
| NM | 5 | 473 | 37.8 | 34.7 | 27.5 |
| Col | 34 | 322 | 37.3 | 46.6 | 16.1 |
| LGD | 14 | 425 | 46.6 | 39.1 | 14.3 |
| HGD | 12 | 498 | 44.8 | 42.2 | 13.0 |
| Ca | 16 | 538 | 52.4 | 39.2 | 8.4 |

The relative loss of telophases in precancerous and cancerous lesions was a most striking result. P, prophase; M, metaphase; T, telophase (addressed by microscopic morphology). Abbreviations as in Table 1.

CDFs and interphase nuclei appears to be a reliable and rapid means for classifying colon adenomas and a rather cheap method for clinical purposes.

### MATERIALS AND METHODS

Specimens were selected from patients of the Flensburg district, Northern Germany. The material comprised normal colon mucosae (five cases), colon adenomas with LGD (14), colon adenomas with HGD (12) and colon adenocarcinomas (16). DNA analysis was performed with mitotic figures and chromosome division figures (CDFs) of matched tissue compartments from which comparative genomic hybridization (CGH) had been carried out previously (Ried et al, 1996). The four microslices (50 μm each) used for CGH were from the hot spot of a lesion. The hot spot was recognized from morphological criteria (Morson, 1985) on the first 5-μm section and was corroborated by high MIB1 decoration on the following 5-μm section. After the CGH sections, two further 5-μm sections were used recording p53 and WAF1 immunoreactivity (Ried et al, 1996). Finally, the penultimate section (8 μm) and the last section (15 μm) were Feulgen stained for interphase and CDF microphotometry respectively. Thus, the distance between morphological diagnosis and microphotometry was at least 215 μm.

In addition, 34 cases of bacterially induced colitis and blood samples from five female and five male individuals, each with a normal karyotype, were used as an external standard. The lymphocytes were stimulated with phytohaemagglutinin in RPMI-1640 medium for 72 h (Moore and Woods, 1976), and spindle formation was inhibited by *N*-methyl-*N*-deacetyl-colchicine (colcemid). After fixation in 4% formaldehyde, our standard Feulgen procedure with hydrolysis in 5 M hydrochloric acid at 22°C for 60 min was applied (Steinbeck et al, 1993).

DNA profiles were generated in 8-μm sections of the CGH compartment, from which 150 interphase nuclei were sampled in each case. Overlapping nuclei and those with degenerated envelopes were precluded by the operator. Cut nuclei were also avoided by focusing up and down searching for gaps. All mitotic

© Cancer Research Campaign 1998

**Figure 2** DNA content from chromosome division figures (CDFs) in 15-μm sections of mucous membranes in colon. HGD in colon adenoma and colon adenocarcinoma revealed DNA amounts beyond the mitotic limit of 4 c. CDFs (P, M and T) in HGD differed significantly from mitotic figures or CDFs in LGD (*t*-test). The number of divisions observed is given in Table 2. Control spreads of 209 prophases of activated lymphocytes (controls) showed 3.99 ± 0.01 c. Abbreviations as in Table 1

**Figure 3** DNA distribution profiles of morphologically addressed metaphases (mad-M) in 15-μm sections. The transition from colitis ($n$ = 150), LGD (166), HGD (210) and Ca (211) was characterized by relative loss of metaphases with plain 4.0 c DNA content figures and CDFs detected were measured in 15-μm sections. CDFs are surrounded with ballooned cytoplasm and were never found overlapped. Cut or degenerated or multipolar CDFs were intentionally excluded. The quantitative records were automatically calibrated against an internal 2 c DNA standard (at least 20 lymphocyte nuclei) and expressed as c-values, where c is the (haploid) genome equivalent. The coefficient of variation (CV) from any lymphocyte population was < 0.07, indicating highly precise measurements (Steinbeck, 1997*b*). DNA profiles matching the mitotic 2–4 c range were obtained from normal colon mucosa and colitis (Figure 1 A) as well as from stimulated lymphocytes (Figure 1 B). The 5 c value provided a sure threshold above which an increased DNA content was observed. As single-cell measurements were performed with high accuracy, the 4.5 c exceeding rate (ER) was used to demonstrate genomic instability.

### RESULTS

### Cell nuclei in interphase

Classification of the adenomas and the degree of dysplasia was confirmed once more by light microscopy, as correct morphological classification and compliance with the CGH approach (Ried et al, 1996) was a prerequisite for this microphotometric analysis. DNA distribution profiles of interphase nuclei from normal mucosa and bacterially induced colitis were compatible with the mitotic cell cycle. As a rule, colitis as well as stimulated lymphocytes showed no 5 c ER in interphase nuclei (Table 1). In contrast, LGD and HGD of colon adenomas showed clearly increased nuclear DNA content when the 5 c ER was > 5%, correlated with a step-by-step reduction of mitotic cells. The main DNA peak of nuclear populations (samples as in Table 1) was 2.02 ± 0.02 c in normal mucosa, 2.11 ± 0.01 c in colitis, 2.02 ± 0.02 c in lymphocytes (Lym), 3.14 ± 0.12 c

**Table 3** DNA measurements in lesions of the colon mucosa related to results from comparative genomic hybridization

| | | Chromosome division figures | | | CGH aberration | |
|---|---|---|---|---|---|---|
| Histology | $n_1$ | 4.5 c ER (%) | 5 c ER (%) | $n_2$ | $n_1$ (%) | Gains and losses[a] |
| NM | 5 | 0 | 0 | 473 | 0 | 0 |
| Col | 34 | 1.0 ± 0.8 | 0.2 ± 0.2 | 322 | ND | ND |
| LGD | 14 | 7.9 ± 2.0 | 2.8 ± 1.3 | 425 | 21 | 0–1 |
| HGD | 12 | 25.8 ± 4.4 | 16.2 ± 4.1 | 498 | 58 | 0–4 |
| Ca | 16 | 62.1 ± 7.1 | 46.3 ± 7.0 | 538 | 88 | 0–14 |

[a]Table 2 in Ried et al (1996). Each entire CDF was measured in each case. NM, normal colon mucosa; Col, bacterially induced colitis; LGD, colon adenoma with low-grade dysplasia; HGD, colon adenoma with high-grade dysplasia; Ca, colon adenocarcinoma; c, genome equivalent (haploid); ER, exceeding rate; $n_1$, number of cases; $n_2$, number of chromosome division figures (CDFs); ND, not determined. Numerical scatter (±) is standard error of the mean. The increase of CDFs was parallelled by increased gains and losses recorded with CGH.

© Cancer Research Campaign 1998 

**Figure 4** Examples of chromosome division figures (CDFs). Col is illustrated in **A–C**, HGD in **D–F** and Ca in **G–I**. Full DNA endoreduplication is recorded in **J–K**. Telophase hemispheres with 2 c DNA in **L** verify that mitotic activity may be observed in HGD. Prophases: **A, D, G, J**; metaphases: **B, E** (upper left corner), **H, K**; telophases: **C, F, I, L**. Bar represents 10 μm

 *© Cancer Research Campaign 1998*

in LGD, 3.54 ± 0.20 c in HGD and 3.92 ± 0.19 c in carcinoma. Typical profiles of nuclear DNA were exemplified. Epithelia of normal mucosa and from colitis displayed a pronounced peak at 2.0 c (Figure 1 A). A 60% fraction of stimulated lymphocytes was shifted to the 4.0 c peak but did not surpass this threshold (Figure 1 B). Aberrant DNA content occurred at a low rate in LGD and at a higher rate in HGD (Figure 1 C and D, Table 1). Increased nuclear DNA content or endoreplication was found in carcinomas in which the mean 5 c ER was 20.9% of total nuclei measured (Figure 1 E).

### Chromosome division figures

Compared with the oral mucosa, the physiologically normal colon is rich in division figures. In total, 2256 events comprising mitotic figures and CDFs were found in morphologically addressed prophases (mad-P), metaphases (mad-M), anaphases and telophases (mad-T); 322 CDFs were observed in bacterially induced colitis, 425 CDFs in LGD, 498 CDFs in HGD and 538 CDFs in invasive carcinoma. Both hemispheres of mad-telophases were counted as a single event. Thus, the frequency of mad-telophases diminished from 16.1% in colitis to 14.3% in LGD. Most striking, however, was the relative drop from 13.0% in HGD to only 8.4% in carcinoma (Table 2). Aberrant CDFs occurred at the onset of LGD and with increased frequency in HGD and carcinoma. In LGD, only a few CDFs were found with variable DNA amounts, whereas the majority of figures showed plain 4.0 c-values as expected for true mitosis.

### DNA content of CDFs

The shift from the mitotic range to aberrant DNA amounts was systematically investigated using single-cell microphotometry. Any mad-CDF detected was quantified for total DNA (Figure 2). CDFs from carcinomas averaged a much higher DNA content (5.4 ± 0.08 c in mad-P, 5.0 ± 0.07 c in mad-M, 5.3 ± 0.17 c of both hemispheres in mad-T) than those from HGD. With regard to mad-P, a mean of 4.1 ± 0.03 c and 4.3 ± 0.05 c was observed in LGD and HGD, respectively. The mean of mad-M was 4.1 ± 0.03 c and 4.4 ± 0.05 c, that of mad-T was 4.0 ± 0.05 c and 4.3 ± 0.09 c in LGD and HGD respectively. The CDFs from the latter lesions deviated significantly from each other (Fisher test for mad-P: 4.53 > $F^*$ = 1.30; mad-M: 5.23 > $F^*$ = 1.32; mad-T: 2.68 > $F^*$ = 1.52; $P = 0.05$ each).

Table 3 summarizes the findings from CDFs in the matched histological compartments from which CGH data had been obtained previously (Ried et al, 1996). As a further DNA standard, 34 cases of colitis were added. In this lesion, 316 of 322 divisions were observed with plain 4 c DNA content and were therefore considered to be truly mitotic figures. One case of colitis showed a focus of four aberrant CDFs (≥ 4.5 c DNA < 5.0 c). In two further cases of colitis, one 4.8 c mad-metaphase and one 5.0 c mad-prophase were found. However, aberrant telophases were not detected in colitis. The 5 c ER of CDFs increased from 2.8% in LGD to 16.2% in HGD and 46.3% in carcinoma. Thus, the spreading of aberrant DNA content engulfed the CDFs to an extent similar to that recorded for interphase nuclei in Table 1. Likewise, an increase in CGH aberrations was observed within the matched compartments, ranging from 21% of cases in LGD and 58% in HGD to 88% in carcinomas (Table 3).

Microphotometry revealed that different cell populations contribute to colon adenomas. Among mother cells and their normally differentiated daughters, there are atypical cells with aberrant CDFs due to an altered DNA content. True mitotic figures were displayed within the 4 c peak in the distribution profiles for colitis, LGD, HGD and carcinoma (Figure 3). Aberrant amounts of nuclear DNA contributed to a progressive flattening of the profiles during tumorigenesis, when DNA values left the 4 c level, shifting beyond the thresholds of 4.5 c and 5 c. As a rule, the increase of the sensitive 4.5 c ER was in line with tumour progression. Those CDFs that deviated to the left side, below 4 c DNA content, demand further investigation (Steinbeck, in preparation). Full reduplication was recorded in only a few CDFs with 8 c DNA content (Figure 4 J and K), but a peak at 8 c did not occur with any lesion in colon epithelia, neither with interphases nor with CDFs.

### Morphology of CDFs

Multipolar aberrations are rare events and were intentionally excluded from this investigation. Nevertheless, quantitative differences were found in the Feulgen DNA of CDFs. Their morphology and microscopic texture resembled (normal) mitotic prophases, metaphases and telophases. Indeed, DNA amounts at the mitotic 4 c level were recorded in the vast majority of divisions in normal mucosa and colitis (Figure 4 A–C). A record of aberrant DNA content (< 4 c and ≫ 4 c) was obtained from CDFs in HGD and, with increasing frequency, in carcinomas (Figure 4 D–I). Notable was the focal appearance of mitoses and CDFs that frequently exhibited synchrony (Figure 4 E, G and L). The CDFs were generally found in the vicinity of endoreplicated interphase nuclei.

Limited morphological evidence for aberrant CDFs was deduced from enlarged chromatin volume and enhanced Feulgen density. However, decisive classification always requires microphotometry.

### DISCUSSION

### Multiple genotype aberrations

Neither gains nor losses have been detected with CGH in genomic DNA of several patients suffering from established cancer (Lundsteen et al, 1995; Ried et al, 1996; Björkqvist et al, 1997). Two interpretations may apply to these negative CGH cases. First, the transforming mechanism may comprise one or more point mutations and would be detected by DNA sequencing only; flow cytometry or image microphotometry would result in so-called 'diploid' DNA profiles. Second, a tumour may contain cells challenged by multiple segregation failures affecting heterologous chromosomes in different nuclei. Scattered amounts of DNA would result in distribution profiles due to DNA aneuploidy (Sandritter, 1966; Hiddemann et al, 1984). Such a situation can be identified only by a quantifying single-cell method, i. e. microphotometry. The scattered DNA amounts observed with interphase nuclei as well as individual CDFs make an exclusive effect of point mutations implausible. Scattering has been observed in cellular records; it is therefore conclusive that clonal selection had not been effective up to the time when CGH trials produced negative results. However, positive microphotometric records show that individual nuclei may be aberrant despite a negative CGH record. Microphotometry can not be used for decisions on point mutations and quantitatively neutral events (inversions and translocations). Microphotometry of CDFs and interphase nuclei in tumours supports clinical discrimination of HGD from LGD. In the present investigation, as material of more than 200 μm depth

© Cancer Research Campaign 1998

was used for CGH, some microphotometric data deviated slightly from the primary diagnosis. However, this type of variation was never observed when slices for microphotometry were taken from areas close to those used for diagnosis (Steinbeck et al, 1993; Steinbeck, 1997 *a* and *b*). Thus, because morphological diagnosis is descriptive and subject to the pathologist's experience, doubtful cases can be resolved by microphotometry. As nuclear mass and volume increase with the power of three, even a trained observer cannot discriminate a factor 2 in DNA content.

### Precision and biological bias

The reliability of microphotometric data depends on whole nuclei retained in sections from paraffin-embedded tissue. Nuclear integrity was achieved in sufficiently deep sections, 8 μm for interphase nuclei and 15 μm for metaphases or CDFs (Steinbeck, 1997 *a* and *b*). Cell-by-cell microphotometry (Caspersson, 1940) has been applied for many tumour sites (Atkin and Richards, 1956). Comparative studies testify to the high accuracy of image microphotometry compared with flow technology (Claud et al, 1989; Askensten et al, 1990; Fausel et al, 1990; Bosari et al, 1992; Steinbeck et al, 1993). High accuracy was also obtained in this investigation from repeated measurements of the same nucleus and by sampling interphase nuclei from healthy tissues; the coefficient of variation (CV) was < 0.03 in normal mucosa. A similarly modest technical scattering, CV = 0.05, was recorded from metaphases in pyogenic granulomas of the oral mucosa (Steinbeck, 1997*b*). Guidelines for flow cytometry of 'normal diploid cells' suggest the CV to be < 0.08 (Shankey et al, 1993). A raised variation, however, provides evidence for biological bias, as recorded from interphase nuclei in bacterially induced colitis (CV = 0.10 within the 1.8–2.4 c fraction). DNA synthesis in the activated mitotic cell cycle would sufficiently explain this observation. However, a much stronger bias became obvious from the fractions of 1.8–2.4 c nuclei in the course of tumorigenesis: CV = 0.62 in LGD, 0.93 in HGD and a dramatic CV = 3.50 in carcinoma (supplementing Table 1).

The heterogeneity of interphase nuclei is referred to as DNA aneuploidy and has remained enigmatic. Any tissue affected with inflammation, e. g. colitis or pyogenic granuloma, displays an activated mitotic cell cycle in which the mitotic figures match the expected amount of 4 c DNA. The CV = 0.05 from prophases, metaphases and telophases in colitis corresponds with mitotic activity. But CDFs from neoplastic lesions clearly abandon DNA constancy, with the observations of CV = 0.11 in LGD, 0.19 in HGD and 0.25 in carcinoma (supplementing Table 3). This increase in numerical scattering demonstrated the genomic instability detected by the microphotometric method, in particular with CDFs.

### Deviation from mitotic regulation

The data from stimulated lymphocytes showed that they followed an activated mitotic cycle. The blood cells did not break the mitotic 2–4 c range, and they did not develop aberrant CDFs. In contrast, the colon epithelia displaying LGD comprised a 4.2% fraction of interphase nuclei that had abandoned the normal genome as monitored by their 5 c ER. The portion of interphase nuclei > 5 c increased, via 10.7% in HGD, to 20.9% in carcinoma, while nuclei of the mitotic range were lost correspondingly. This led to the conclusion that colonic epithelia had already met in LGD genomic disturbances that were caused by the transformation trigger, probably point mutation(s), leading to malignant clone(s) (Nowell, 1976; Prehn, 1994).

The record of CDFs surprisingly revealed DNA profiles resembling those from the more frequent interphase nuclei in the respective lesions (Figure 1C, LGD; Figure 1 D, HGD). Corresponding to tumour progression, the 5 c ER of CDFs increased from 2.8% in LGD, via 16.2% in HGD, to 46.3% in carcinoma. Because of the significant bearing of numerical scattering and the high precision obtained from normal tissue, the more sensitive 4.5 c ER was added (Table 3), revealing the aberrations of somatic nuclei during tumorigenesis. The aberrant DNA content could be generated by local amplification or incomplete endoreplication in interphase and/or by non-disjunction in anaphase, because the data did not match a geometric $2^n$ increase.

### DNA replication released from mitosis

The interphase nuclei and CDFs at 8 c and higher levels provide further evidence that the components of the mitotic event need tight genetic regulation for their normally synergistic function. Spindle formation, karyokinesis and cytokineses may proceed in the absence of chromosomes (Heald et al, 1996; Zhang and Nicklas, 1996). The fact that uncoupling of S-phase from mitosis produces endoreplicated nuclei has been known for a long time (Nagl, 1978; Waldmann et al, 1996). After one or more additional DNA syntheses in the nucleus, the appropriate genetic signal may be expressed or may accumulate above threshold, triggering condensation of the endoreplicated chromatin. Endomitosis makes the multiplied chromosomes divide in wild-type organisms without a spindle and without dissociation of the nuclear membrane (Geitler, 1949). In CDFs however, the condensation signal does not always wait for perfect DNA reduplication but may become effective autonomously as seen from colon mucosa (Figure 3) and oral mucosa (Steinbeck, 1997*a*). CDFs obviously also condense their chromatin below plain 4 c. In particular the unequal hemispheres (mad-telophases; Figure 4 F and I) made apparent multiple chromosomal aberrations in tumorigenesis.

### Genomic instability and unequal telophases

Timonen and Therman (1950) have observed a shift of the prophase–metaphase ratio during cancer development. The relative loss of mad-telophases (Table 2) leads to the conclusion that there is strong selection against CDFs. This genetic checkpoint at the transition from metaphase through anaphase to telophase may represent a vital defence mechanism, as unequal DNA amounts have frequently been recorded from the two halves of a mad-telophase. When such hemispheres escape selection and obtain proliferative ability, genomic instability is finally established. It appears likely that these unequal telophases bring about cytogenetic (chromosomal) aneuploidy (Mitelman, 1994; Heim and Mitelman, 1995). Those mad-metaphases below 4.0 c are either defective daughters of unequal mad-telophases, or they represent instances of chromatin condensation before DNA replication was completed.

### CONCLUSION

Tumorigenesis appears as a multistep process not only in molecular terms (Vogelstein, 1993) but also in morphological alterations: disturbance of mitotic differentiation, increase of nuclear DNA and

© Cancer Research Campaign 1998

accumulation of CDFs (Steinbeck, 1997*b*). The CDFs characterize not only the final cancer but are already apparent in precancerous conditions. Many CDFs enhance the probability of unequal anaphases and telophases. In the cascade of transformation, aberrant telophases may meet positive clonal selection, after which a prominent nuclear fraction should comprise an aberrant genome. If genomic DNA is isolated after clonal selection, CGH can detect these generalized gains and losses. CGH investigation before the clonal event may fail to detect genomic deviations. However, single-cell microphotometry reveals a chaotic nuclear quake occurring early and spreading more and more during tumorigenesis.

### ACKNOWLEDGEMENTS

Professor Gert Auer, Department of Oncology and Pathology, Karolinska Institute, Stockholm, provided encouragement and some of the research facilities. Mr Uwe Koester, Flensburg, gave excellent technical assistance. Human lymphocyte specimens were courtesy of Professor Horst Hameister, Ulm. Statistics and critical comments were from Dr rer. nat. Helmut Zacharias, Langwedel. Ms Jayne Welling-Wolf, Kiel, Germany, provided linguistic aid. This study was supported by the Swedish Cancer Society and by the Research Funds of the Institute of Pathology, Flensburg, Germany.

### REFERENCES

Askensten UG, Moberger B and Auer GU (1990) Methodological aspects on cytochemical DNA assessments of adenocarcinoma in the endometrium by means of image and flow cytometry using conventional formalin-fixed and paraffin-embedded specimens. *Arch Geschwulstforsch* **60**: 209–216

Atkin NB and Richards BM (1956) DNA in human tumours as measured by microspectrophotometry of Feulgen stain: a comparison of tumours arising at different sites. *Br J Cancer* **10**: 769–786

Björkqvist A-M, Tammilehto L, Anttila S, Mattson K and Knuutila S (1997) Recurrent DNA copy number changes in 1q, 4q, 6q, 9p, 13q, 14q and 22q detected by comparative genomic hybridization in malignant mesothelioma. *Br J Cancer* **75**: 523–527

Bosari S, Lee AKC, Wiley BD, Heatley GJ, Hamilton WM and Silverman ML (1992) DNA quantitation by image analysis of paraffin-embedded colorectal adenocarcinomas and its prognostic value. *Modern Pathol* **5**: 324–328

Brodsky VY and Uryvaeva IV (1985) *Genome Multiplication in Growth and Development: Biology of Polyploid and Polytene Cells*. Cambridge University Press: Cambridge

Caspersson T (1940) Methods for the determination of the absorption spectra of cell structures. *J R Microscop Soc* **60**: 8–25

Claud RD, Weinstein RS, Howeedy A, Straus AK and Coon JS (1989) Comparison of image analysis of imprints with flow cytometry for DNA analysis of solid tumors. *Modern Pathol* **2**: 463–467

Fausel RE, Burleigh W and Kaminsky DB (1990) DNA quantification in colorectal carcinoma using flow and image analysis cytometry. *Anal Quant Cytomet Histol* **12**: 21–27

Geitler L (1949) Ergebnisse und Probleme der Endomitoseforschung. *Österr Bot Ztschr* **95**: 277–299

Hansemann D (1890) Ueber asymmetrische Zelltheilung in Epithelkrebsen und deren biologische Bedeutung. *Arch Pathol Anat Physiol Klin Med* **119**: 299–326

Heald R, Tournebize R, Blank T, Sandaltzopoulos R, Becker P, Hyman A and Karsenti E (1996) Self-organization of microtubules into bipolar spindles around artificial chromosomes in Xenopus egg extracts. *Nature* **382**: 420–425

Heim S and Mitelman F (1995) *Cancer Cytogenetics*. 2nd edn. Wiley-Liss: New York

Heitz E (1953) Über intraindividuale Polyploidie. *Arch Jul Klaus-Stiftung Vererbungsforsch Sozialanthropol Rassenhyg* **28**: 260–271

Hiddemann W, Schumann J, Andreeff M, Barlogie B, Herman CJ, Leif RC, Mayall BH, Murphy RF and Sandberg AA (1984) Convention on nomenclature for DNA cytometry. *Cytometry* **5**: 445–446

Kallioniemi A, Kallioniemi O-P, Sudar D, Rutovitz D, Gray JW, Waldman F and Pinkel D (1992) Comparative genomic hybridization for molecular cytogenetic analysis of solid tumors. *Science* **258**: 818–820

Lundsteen C, Maahr J, Christensen B, Bryndorf T, Bentz M, Lichter P and Gerdes T (1995) Image analysis in comparative genomic hybridization. *Cytometry* **19**: 42–50

Mitelman F (1994) *Catalog of Chromosome Aberrations in Cancer*. 5th ed. Wiley-Liss: New York

Moore GE and Woods LK (1976) Culture media for human cells: RPMI 1603, RPMI 1634, RPMI 1640 and GEM 1717. *Tissue Culture Association Manual* **3**: 503–508

Morson BC (1985) Precancer and cancer in inflammatory bowel disease. *Pathology* **17**: 173–180

Nagl W (1978) *Endopolyploidy and Polyteny in Differentiation and Evolution*. Elsevier: Amsterdam

Nowell PC (1976) The clonal evolution of tumor cell populations. *Science* **194**: 23–28

Prehn RT (1994) Cancer beget mutations versus mutations beget cancer. *Cancer Res* **54**: 5296–5300

Ried T, Knutzen R, Steinbeck R, Blegen H, Schröck E, Heselmeyer K, Du Manoir S and Auer G (1996) Comparative genomic hybridization reveals a specific pattern of chromosomal gains and losses during the genesis of colorectal tumors. *Genes Chromosomes Cancer* **15**: 234–245

Sandritter W (1966) Methods and results in quantitative cytochemistry. In *Introduction to Quantitative Cytochemistry*, Vol. 1, Wied GL. (ed.), pp. 159–181. Academic Press: New York

Shankey TV, Rabinovitch PS, Bagwell B, Bauer KD, Duque RE, Hedley DW, Mayall BH and Wheeless L (1993) Guidelines for implementation of clinical DNA cytometry. *Cytometry* **14**: 472–477

Steinbeck RG (1997*a*) Proliferation and DNA aneuploidy in mild dysplasia imply early steps of cervical carcinogenesis. *Acta Oncol* **36**: 3–12

Steinbeck RG (1997*b*) Atypical mitoses in lesions of the oral mucosa: a new interpretation of their impact upon tumorigenesis. *Oral Oncol* **33**: 110–118

Steinbeck RG, Heselmeyer KM, Neugebauer WF, Falkmer UG and Auer GU (1993) DNA ploidy in human colorectal adenocarcinomas. *Anal Quant Cytol Histol* **15**: 187–194

Therman E, Sarto GE and Kuhn EM (1986) The course of endomitosis in human cells. *Cancer Genet Cytogenet* **19**: 301–310

Timonen S and Therman E (1950) The changes in the mitotic mechanism of human cancer cells. *Cancer Res* **10**: 431–439

Vogelstein B (1993) Genetic alterations in colorectal tumors. *Adv Oncol* **7**: 3–6

Waldman T, Lengauer C, Kinzler KW and Vogelstein B (1996) Uncoupling of S phase and mitosis induced by anticancer agents in cells lacking p21. *Nature* **381**: 713–716

Zhang D and Nicklas RB (1996) 'Anaphase' and cytokinesis in the absence of chromosomes. *Nature* **382**: 466–468

© Cancer Research Campaign 1998